# Editorial: Multisensory integration as a pathway to neural specialization for print in typical and dyslexic readers across writing systems

**DOI:** 10.3389/fpsyg.2022.992380

**Published:** 2022-08-17

**Authors:** Susana Araújo, Urs Maurer, Tânia Fernandes

**Affiliations:** ^1^CICPSI, Faculty of Psychology, University of Lisbon, Lisbon, Portugal; ^2^Department of Psychology and Brain and Mind Institute, The Chinese University of Hong Kong, Hong Kong, Hong Kong SAR, China

**Keywords:** visual-orthographic system, audiovisual integration, motor-visual integration, dyslexia, training

Active participation as a citizen depends on fluent decoding and production of written language. Efficient processing of graphs^1^ is the foundation of reading (Pelli et al., 2003), as graphs are the building blocks of written words from early on in reading acquisition to skillful reading (Grainger, 2018). How does the human brain become specialized and process graphs and written words in the context of the multimodal nature of the reading experience? This is the focus of this Research Topic. It includes a Research Topic of 13 articles that cover current issues in the cognition and neurobiology of reading development and variability. Groups with a wide range of reading skills took part in these studies, and various behavioral tests and neuroimaging techniques (EEG-ERPs, fMRI) were used to investigate how learning audio-visual and motor-visual associations relate to (in)efficient graph recognition and reading across alphabetic and logographic writing systems.

This Research Topic begins with two studies focusing on orthographic processing (i.e., encoding of information about letter identities and letter positions), a key interface between low-level visual processes and higher-level processing of words during reading (Grainger, 2018). Fernández-López et al. investigated the early precursors of precise letter position coding in pre-schoolers via the transposed-letter effect, i.e., failing to efficiently differentiate between CHOLOCATE and CHOCOLATE. Results highlighted ^1^ that learning to read is built also on a basic cognitive foundation, by showing that sequential memory and perception skills shape pre-readers' ability to encode letter position accurately (reflected in the size of the transposed-letter effect in a same-different task: TZ-ZT vs. TZ-TZ, previously reported in Perea et al., 2016). The importance of orthographic processing for reading development is also reflected in the longitudinal study by Eberhard-Moscicka et al. They investigated 1st-grade children with EEG and tested reading skills in the same children 3 years later. *N1 print tuning*, measured as an N1 increase in response to words compared to false-font strings, together with the mismatch negativity (MMN) improved the prediction of future reading skills compared to behavioral measures alone (RAN, vocabulary, and block design).

The second part of this Research Topic comprises six studies investigating letter-speech sound integration as an emergent property of learning to read. In a review article, Romanovska and Bonte offer a comprehensive picture of the brain basis of reading and a unifying framework with a developmental, dynamic skill learning perspective. They consider the shift from preliterate speech processing to the reading processes in the literate brain, and how dorsal spoken language and the ventral visual brain networks are gradually shaped, by the incremental development of phonological and orthographic knowledge, into an integrated audio-visual reading network. Karipidis et al. provided empirical longitudinal evidence of reading skill-dependent development, from pre-reading to the 1st and 2nd grades, in the functional activity of the superior temporal gyrus (STG), inferior frontal gyrus (IFG), and vOTC during audiovisual processing of single letter-speech sound correspondences.

In turn, four articles provided evidence regarding the audiovisual integration of single characters (in Latin alphabet and Chinese Calabrich et al.; Fraga-González et al.; Xia et al.) and of letter strings and spoken words (Varga et al.) by dyslexic readers. Specifically, Xia et al. provided evidence that the IFG and STG regions are also involved in the audiovisual processing of morpho-syllabic Chinese. While audiovisual integration effects in these regions did not differ between children with and without dyslexia for Chinese characters, a different correlational pattern of these effects with cognitive measures suggested that different neurocognitive networks shape the integration effects in children with and without dyslexia. Moreover, the same study also found a different audiovisual integration pattern for alphabetic pinyin compared to characters, which may reflect the specific role of pinyin as a scaffolding mechanism for learning Chinese characters. In an eye-tracking study, Calabrich et al. showed that adults with dyslexia recognized and recalled fewer newly learned letter-speech sound bindings than control readers. Dyslexics also showed an overreliance on (seemingly irrelevant) episodic cues during stimulus exposure to aid memory retrieval, specifically on the consistency of contextual stimulus properties, which “may be indicative of a more fragile memory representation” (p. 12). Fraga-González et al. adopted a graph theoretical approach for assessing EEG activity in dyslexic and typical readers during an artificial audiovisual learning task. Dyslexic were as able as control adults to accurately learn the novel bindings (i.e., no behavioral group difference), but showed lower theta connectivity during task performance and lower theta degree correlation over task and rest recordings, suggesting reduced (long distance) network integration and less communication between network nodes compared to typical readers. Finally, at the word level, using an implicit same-different perceptual-matching task, Varga et al. found that, whilst reading groups did not differ in ERP correlates of letter identity and letter position encoding in the visual modality, only typical adult readers but not those with dyslexia seemed to show automatic phonological processing and audiovisual integration when the visual letters and speech sounds were presented simultaneously (i.e., larger N1 responses to words than to pseudowords when orthographic stimuli were presented audiovisually).

The last part of this Research Topic focused on the other cross-modal binding promoted when learning to read, that is, between the visual representation of graphs and the corresponding writing gestures. It has been demonstrated that handwriting training during learning of visual graphs is more beneficial for subsequent visual graph recognition than are other learning experiences (e.g., viewing only, typewriting; for a recent meta-analysis, see Araújo et al., 2022). In an opinion article, Fernandes and Araújo reviewed and discussed the available evidence and the three theoretical proposals regarding the underlying mechanism(s) underpinning this handwriting benefit and proposed new directions to disentangle and investigate them. Seyll and Content provided empirical evidence in preschool children for the proposal that detailed visual analysis, which is inherent to handwriting, could be the key to the benefit of this learning experience in subsequent visual graph recognition rather than the graphic motor programs *per se*. Guan et al. showed that, relative to a view-only control training, the contribution of handwriting to visual word recognition also holds in a non-alphabetic script, both at behavioral and electrophysiological levels. Children with dyslexia did not benefit from such a multisensory graph integration. Note, however, that this conclusion might be premature, given that participants were engaged in handwriting for a few seconds and in a single exposure.

Vinci-Booher and James investigated the developmental trajectory of the neural system supporting handwriting, by contrasting fMRI, BOLD-signal change in children and adults during handwriting, and two sensorimotor control tasks. The results indicated that ventral-temporal involvement during handwriting may be adult-like by as early as 5–8 years of age, but a dorsal neural system including the more anterior parietal and frontal motor regions (related to the execution of the motor action) may still be developing in young children at the earliest stages of learning to read. A positive correlation further indicated that the response in these dorsal motor regions during handwriting may be related to children's emerging literacy skills (i.e., letter-word identification).

Finally, Fischer and Luxembourger addressed the challenge of mirror-image discrimination that beginners face when learning to read and write (e.g., b is different than d). These authors tested three candidate models for explaining the almost systematic reversal errors (e.g., b-d) found in writing by learners, using the data made publicly available by Torres et al. (2021). Given that none of these models convincingly accounted for this evidence, the authors suggest that reversal errors may result from a process of symmetrization, achieved through the homotopic interhemispheric exchange in the formation of memory circuits (Corballis, 2018) which may also be determined by the graphs themselves, specifically by the dynamics of writing letters.

This Research Topic provides an exciting overview of the importance of multi-systems interplay during reading development. This collection of papers illustrates the diversity of approaches in this research topic, from experimental psychology and cognitive and clinical neurosciences, adopting different paradigms, combining behavioral and neuroimaging tools, and testing different populations, such as beginning readers, typically-developing and dyslexic readers, alphabet and Chinese literate. It will also hopefully prompt and inspire new questions and directions in reading research in the context of the multimodal experience of reading which bridges visual, auditory, and motor brain systems.

## Author contributions

SA, UM, and TF conceptualized together this Research Topic, critically revised the Editorial, and prepared the revised version. SA has the first authorship and wrote the first draft of the manuscript. All authors approved the final version submitted and the revised version.

## Funding

The preparation of this Research Topic was supported by the Portuguese Foundation for Science and Technology, FCT (Ref. PTDC/PSI-GER/3281/2020 and Ref. 2021.03462.CEECIND).

## Conflict of interest

The authors declare that the research was conducted in the absence of any commercial or financial relationships that could be construed as a potential conflict of interest.

## Publisher's note

All claims expressed in this article are solely those of the authors and do not necessarily represent those of their affiliated organizations, or those of the publisher, the editors and the reviewers. Any product that may be evaluated in this article, or claim that may be made by its manufacturer, is not guaranteed or endorsed by the publisher.

## Footnotes

^1^We adopt the term “graph” to refer to characters of a written script which code linguistic units as phonemes or syllables, such as letters or aksharas.

## References

[B1] Araújo S. Domingues M. Fernandes T. (2022). From hand to eye: a meta-analysis of the benefit from handwriting training in visual graph recognition. Educ. Psychol. Rev. 10.1007/s10648-021-09651-4

[B2] Corballis M. C. (2018). Mirror-image equivalence and interhemispheric mirror-image reversal. Front. Hum. Neurosci. 12, 140. 10.3389/fnhum.2018.00140 PMC5907058 29706878

[B3] Grainger J. (2018). Orthographic processing: a “mid-level” vision of reading: the 44th Sir Frederic Bartlett Lecture. Q. J. Exp. Psychol. 71, 335–359. 10.1080/17470218.2017.1314515 28376655

[B4] Pelli D. G. Farell B. Moore D. C. (2003). The remarkable inefficiency of word recognition. Nature. 423, 752-756. 10.1038/nature01516 12802334

[B5] Perea M. Jiménez M. Gomez P. (2016). Does location uncertainty in letter position coding emerge because of literacy training? J. Exp. Psychol. Learn. Mem. Cogn. 42, 996–1001. 10.1037/xlm0000208 26651460

[B6] Torres A. R. Mota N. B. Adamy N. Naschold A. Lima T. Z. Copelli M.. (2021). Selective inhibition of mirror invariance for letters consolidated by sleep doubles reading fluency. Curr. Biol. 31, 742-752. 10.1016/j.cub.2020.11.031 33338430

